# Differences in Water Consumption of Wheat Varieties Are Affected by Root Morphology Characteristics and Post-anthesis Root Senescence

**DOI:** 10.3389/fpls.2021.814658

**Published:** 2022-01-31

**Authors:** Xuejiao Zheng, Zhenwen Yu, Yu Shi, Peng Liang

**Affiliations:** National Key Laboratory of Crop Biology, Agronomy College, Shandong Agricultural University, Tai’an, China

**Keywords:** winter wheat, yield potential, water use, root morphology, root senescence

## Abstract

Selecting high-yielding wheat varieties for cultivation can effectively increase water use efficiency (WUE) in the Huang–Huai–Hai Plain, where is threatened by increasing water shortages. To further identify the difference in water use and its relationship with root morphology and senescence characteristics, wheat varieties with different yield potentials—Yannong 1212 (YN), Jimai 22 (JM), and Liangxing 99 (LX)—were studied in a high-yielding wheat field. The water consumption percentage (CP) in YN decreased from planting to anthesis; however, crop evapotranspiration and CP increased from anthesis to maturity compared with JM and LX. In YN, a higher soil water consumption from anthesis to maturity in the 0–100 cm soil layer was partly attributed to the greater root weight density in the 20–60 cm soil layer. In topsoil (0–40 cm), root length density, root surface area density, and root diameter at 20 days after anthesis, root superoxide dismutase activity, and root triphenyl tetrazolium chloride reduction activity during mid grain filling stage were higher in YN than in JM and LX. YN had the highest grain yields of 9,840 and 11,462 kg ha^–1^ and increased grain yield and WUE by 12.0 and 8.4%, respectively, as compared with JM, and by 30.3 and 21.3%, respectively, as compared with LX. Ensuring more soil water extraction post-anthesis by increasing roots in the 20–60 cm soil profile, improving root morphology traits, and alleviating root senescence in the topsoil during mid-grain filling stage will assist in selecting wheat varieties with high yield and WUE.

## Introduction

The Huang–Huai–Hai Plain (3HP), as the main wheat production region in China, owns only 40% of the agricultural land in the country; however, it accounts for about 61% of the total wheat production of the country (Sun et al., 2011; Xu et al., 2018). In this area, the precipitation received during the full wheat growth season ranges from 100 mm to 180 mm, which can only meet 25–40% of the total water requirement of wheat (Iqbal et al., 2014). Groundwater is used on 64% of the irrigated area, and excessive extraction has led to a rapid decline in groundwater table (Zhang et al., 2010). Improving water use efficiency (WUE) is a crucial approach for food security in regions such as the 3HP, where water shortage is a major challenge for agriculture (Brauman et al., 2013; Rashid et al., 2019). Therefore, reasonable irrigation strategies have been proposed (i.e., regulating irrigation frequency and timing, applying deficit irrigation, and supplemental irrigation) for increasing WUE and maintaining high wheat production (Wang, 2017; Meena et al., 2019a; Zhao et al., 2020). However, studies have shown that renewing varieties is an effective way to increase yield and WUE (Faralli et al., 2019; Meena et al., 2019b), and it is necessary to study the physiological characteristics of wheat varieties with high yield and high WUE.

The variation of wheat varieties in yield and WUE has been reported in some studies (Zeleke and Nendel, 2016; Ma’arup et al., 2020). When wheat yield ranging from 6,475 to 7,275 kg ha^–1^ under the irrigation of 190 and 230 mm in two wheat varieties, using a greater yield and WUE wheat variety Shijiazhuang 8 could increase the WUE up to 17.8 kg ha^–1^ mm^–1^ by reducing the irrigation amount without decreasing the yield (Liu et al., 2016). Zhang et al. (2010) reported that the WUE of wheat is estimated to increase from 10.0 to 12.0 kg ha^–1^ mm^–1^ for the early 1970s varieties to 14.0–15.0 kg ha^–1^ mm^–1^ for the 2000s varieties in the 3HP, and the positive relationship between grain yield and WUE for all the varieties indicated that using a higher-yielding variety has the potential to improve WUE, thereby saving water. However, limited information is available on the variation of WUE in water consumption characteristics between varieties with different yield levels during the entire growing season. There is a need to develop high-yielding wheat varieties because achieving greater yield from the same water resources contributes to higher WUE (Zhang et al., 2017).

Increased grain yield could be achieved by breeding deeper-rooted wheat varieties in specific farming systems as root distribution in the soil is directly associated with soil water uptake (Wasson et al., 2012; Severini et al., 2020). In rice, increased root length density is required for achieving high crop yield and WUE (Yang et al., 2012), and inadequate root length could accelerate the senescence of above-ground plants during the grain filling stage (Liu et al., 2018). However, Fang et al. (2021) reported that in the semi-arid region on the Loess Plateau, modern wheat varieties with yields between 5,814 and 6,115 kg ha^–1^ had less root biomass and root length density in the 0–40 cm soil layer, which reduced pre-anthesis water uptake but increased soil water extraction after anthesis, contributing to the increases in grain yield and WUE. Regulating root morphology characteristics by irrigation technologies can influence the uptake of soil water during the post-anthesis phase, that have major effects on wheat yield and WUE (Ali et al., 2019). However, the onset of wheat senescence during post-anthesis is inevitable (Nehe et al., 2020). Guaranteeing wheat water uptake during this phase can provide important advantages in yield formation (Li et al., 2019). Much attention has been paid to the effects of root morphology characteristics on crop yield and WUE; however, little information is available about the root senescence traits post-anthesis and its relationship with the soil water uptake and grain yield of wheat varieties with different yield levels.

Wheat varieties have been replaced 8–9 times in the main wheat production regions of China since the 1950s, with great improvement in yield potential (He et al., 2018). The average wheat yield in Shandong, Henan, and Hebei Provinces, as the main wheat production regions in the 3HP, varied from 6,052.5 to 6,484 kg ha^–1^ from 2014 to 2017 (Ministry of agriculture and rural affairs of the People’s Republic of China, 2016, 2019). Widely planted winter wheat varieties, Jimai 22 (JM) and Liangxing 99 (LX), in the 3HP are important parental resources for the current wheat variety improvement in China (Yang Z. Z. et al., 2020). The well-adapted wheat variety, Yannong 1212 (YN), has broken the yield record of winter wheat twice in China and showed a high yield of over 12,000 kg ha^–1^ at eight different locations in the 3HP since 2015 (Dazhong Daily, 2020). The recorded grain yield of YN was 11,001 kg ha^–1^, which was 19.5% and 34.4% higher than the varieties JM and LX in a previous study (Liang et al., 2018). However, few studies have been conducted to investigate the differences in water consumption characteristics, root morphology, and senescence traits, underlying yield superiority of YN to JM and LX. Moreover, clarifying the difference in water use of wheat varieties and its relationships with root morphology and senescence characteristics may also contribute to the improvement of new varieties with high yield and high WUE in future breeding programs. The objectives of this study were to: identify the water consumption characteristics associated with high-yielding and high WUE wheat varieties and clarify the relationships among root morphology and post-anthesis senescence characteristics, water use, and wheat production.

## Materials and Methods

### Experimental Site

Field experiments were carried out from 2017 to 2019 in Shijiawangzi village, Yanzhou (116°41′E, 35°42′N), Shandong Province, China. This region has a typical and representative environment of the 3HP. The average annual groundwater depth is 25 m, and the soil type is Haplic luvisol (FAO classification system). Before sowing, the soil layer (0–20 m) contained 19.2 g kg^–1^ organic matter, 1.2 g kg^–1^ total nitrogen (N), 166.3 mg kg^–1^ hydrolyzable N, 56.2 mg kg^–1^ available phosphorus, and 204.3 mg kg^–1^ available potassium. The soil bulk density and field capacity in the 0–200 cm soil layer before sowing are shown in Supplementary Table 1. Precipitation during the wheat-growing seasons in 2017–2018 and 2018–2019 are shown in Supplementary Figure 1.

### Experimental Design

Seeds of three winter wheat varieties with different yield potentials—YN, JM, and LX—were used in the present study. Among the three wheat varieties, YN was approved by National Crop Variety Approval Committee in 2020 (Ministry of agriculture and rural development of the People’s Republic of China, 2020). JM and LX have been sown in a cumulative area of 60 million ha due to the excellent performance in wheat production. JM (since 2015) and LX (from 2010 to 2015) were successively employed as control varieties in the performance test of new cultivars in the 3HP (Yang Z. Z. et al., 2020). A randomized design with three replications was implemented. Each experimental plot was 2 m × 30 m in size with a 2.0 m buffer zone between plots to minimize the effects of adjacent plots. The straw of the preceding maize crop was crushed and returned into the cropland. A base fertilizer of 105 kg ha^–1^ N, 150 kg ha^–1^ P_2_O_5_, and 150 kg ha^–1^ K_2_O were applied before sowing, and at the jointing stage of wheat production, 165 kg ha^–1^ N was ditched. Wheat seeds were sown on October 23, 2017, and October 10, 2018, with a population of 270 plant m^–2^ and 180 plant m^–2^, respectively, while wheat plants were harvested on June 7, 2018, and June 11, 2019. Experimental plots were irrigated with 60 mm of water each in the pre-winter, jointing, and anthesis stages, totaling 180 mm of water during the growing season.

### Sampling and Measurements

#### Soil Water Content and Crop Evapotranspiration

In all experimental plots, soil from the surface to 200 cm depth was sampled at 20 cm intervals using a soil corer. Measurements of soil samples were taken at pre-planting, a day before irrigation at both the jointing and anthesis stages, and at maturity from each treatment with three replicates. The soil water content of each 20 cm soil layer was determined by using the oven-drying method described by Gardner (1986).

The crop water consumption or crop evapotranspiration (ET) of winter wheat for the whole growing season or an individual growth period was based on the equation described by Chattaraj et al. (2013):


ET=Δ⁢SW+I+P-D-R

Where ET (mm) is the crop evapotranspiration, _Δ_ SW (mm) is the soil water consumption of 0–200 cm soil profile between two specific growth stages, I (mm) is the irrigation amount, P (mm) is the precipitation amount, D (mm) is downward flux below the crop root zone, and R (mm) is surface runoff. In this experimental site, owing to the presence of a low groundwater table (average annual of 25 m) below the ground surface, capillary rise, drainage, and runoff were not calculated. Drainage and runoff are negligible in the 3HP, including in this experimental site (Lv et al., 2011).

The water consumption percentage (CP) for a given growth period was the ratio of the water consumption amount or ET of that period to the total crop evapotranspiration during the whole growth periods of wheat (ET_C_) (Xu et al., 2018).

### Root Weight Density

Roots were sampled from each treatment with three replicates at anthesis, using an auger with a 10 cm internal diameter, at 20 cm intervals down to a 100 cm depth. At each plot, two cores were collected within and between the wheat rows. The roots from the core sections, with mixtures of roots and soil in each 20 cm soil layer, were collected following the method described by Jha et al. (2017). Root samples were oven-dried at 80°C until a constant weight was reached. The root weight density (RWD) was calculated according to Feng et al. (2017).

### Root Morphology Characteristics and Biochemical Assays

Root samples in the 0–40 cm soil layer were collected at 20 cm intervals from each pot with three replicates at anthesis, 10 and 20 days after anthesis (DAA), using an auger with a 10 cm internal diameter. Each sample included two cores that collected within and between the wheat rows. Root samples were collected and refrigerated at −80°C. Half of the root samples were used to measure root length, root surface area, and root diameter measurements using an Epson V700 scanner (Seiko Epson Corp., Japan) and WinRHIZO 2013 software (Regent Instruments Canada Inc., Canada). Root length density and root surface area density were calculated according to He et al. (2019). The remaining root samples were used for the measurements of root malondialdehyde (MDA) concentration, superoxide dismutase (SOD) activity, and root activity. Root MDA concentration and SOD activity were assayed using the methods described by Guo et al. (2015). Root activity was determined following the method of Man et al. (2016) and represented by the triphenyl tetrazolium chloride (TTC) reduction activity.

### Grain Yield

At maturity, grain yield was determined by the plants harvested from a 5 m^2^ area in each plot and sun-dried to 12.5% moisture content before being recorded.

### Water Use Efficiency

The WUE was calculated as follows (Ma et al., 2019):


W⁢U⁢E=Y/E⁢T⁢c

Where *WUE* (kg ha^–1^mm^–1^) represents the crop water use efficiency, *Y* (kg ha^–1^) is the grain yield, and *ET*_*C*_ (mm) is the total crop evapotranspiration during the whole growing periods of wheat.

### Statistical Analysis

Statistical analysis was performed using standard ANOVA in SPSS 22.0 (IBM, New York, NY, United States). The normality of data and the homogeneity of variances were checked by using the Shapiro–Wilk test and Levene’s test, respectively. ANOVA was conducted to compare the effects of different treatments on the measured variables. The means were compared using Duncan’s test at α = 0.05 to identify significant effects.

## Results

### Crop Evapotranspiration in Different Growth Periods

As shown in Table 1, the ET_C_ of YN was significantly higher than that of JM and LX in both years. There was no significant difference in ET from planting to jointing stages in all wheat varieties in 2017–2018. Compared with JM and LX, the CP from planting to jointing of YN was 4.9 and 7.8% lower in 2017–2018, and 5.6 and 8.1% lower in 2018–2019, respectively. ET from jointing to anthesis did not differ significantly among YN, JM, and LX, despite that YN had the lowest CP from jointing to anthesis. ET and CP from anthesis to maturity, both ranked in the decrease order of YN > JM > LX, in both years.

**TABLE 1:** Total crop evapotranspiration during the whole growing periods of wheat (ET_C_), crop evapotranspiration, and water consumption percentage in different growth periods in the 2017–2018 and 2018–2019 growing seasons.

Year	Treatments	ET_C_	Planting-Jointing		Jointing-Anthesis		Anthesis-Maturity	
		(mm)	ET (mm)	CP (%)	ET (mm)	CP (%)	ET (mm)	CP (%)
2017–2018	YN	546.2a	193.1a	35.35c	140.2a	25.67b	212.9a	38.98a
	JM	522.8b	194.4a	37.18b	142.4a	27.24a	186.0b	35.58b
	LX	499.6c	191.4a	38.32a	139.9a	28.00a	168.3c	33.68c
2018–2019	YN	658.4a	168.6b	25.61b	171.6a	26.07b	318.1a	48.32a
	JM	644.8b	175.0a	27.14a	174.3a	27.04a	295.4b	45.82b
	LX	623.8c	173.8ab	27.87a	172.0a	27.57a	278.0c	44.56c

*YN, Yannong 1212; JM, Jimai 22; LX, Liangxing 99; ET, crop evapotranspiration; CP, water consumption percentage. Values followed by a different letter are significantly different (Duncan’s test, p < 0.05) within the treatments in each year.*

### Soil Water Consumption in Different Growth Periods

**FIGURE 1:**
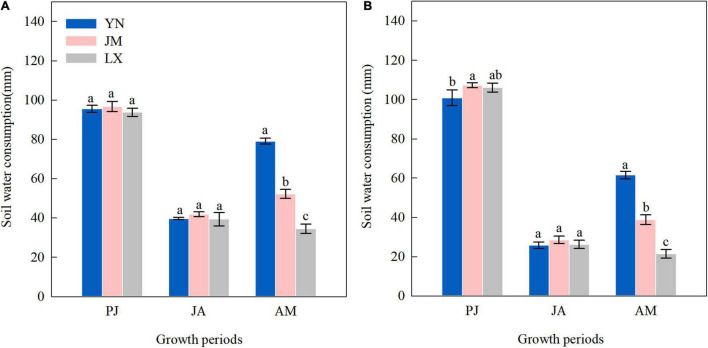
Soil water consumption in different growth periods of wheat varieties in the 2017–2018 **(A)** and 2018–2019 **(B)** growing seasons. YN, Yannong 1212; JM, Jimai 22; LX, Liangxing 99; PJ, planting to jointing; JA, jointing to anthesis; AM, anthesis to maturity. The different letters in the figure indicate significant differences (Duncan’s test, *p* < 0.05). Vertical bars represent the standard deviation of the means.

The soil water consumption from planting to jointing of YN was lower than that of JM in 2018–2019 (Figure 1). The soil water consumption from jointing to anthesis among the three varieties did not differ significantly in both years. The soil water consumption values from anthesis to maturity in YN were 79.1 mm and 61.6 mm in 2017–2018 and 2018–2019, respectively, which were significantly higher than that of JM and LX in both years.

### Soil Water Consumption in the 0–200 cm Soil Layer From Anthesis to Maturity

**FIGURE 2:**
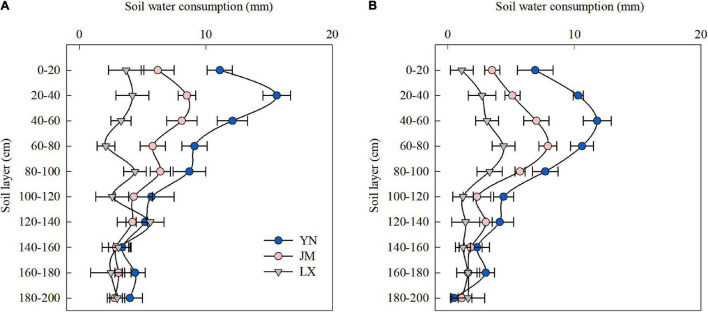
Soil water consumption in the 0–200 cm soil layer from anthesis to maturity of wheat varieties in the 2017–2018 **(A)** and 2018–2019 **(B)** growing seasons. YN, Yannong 1212; JM, Jimai 22; LX, Liangxing 99. Vertical bars represent the SD of the means.

The highest soil water consumption in the 0–100 cm soil layer from anthesis to maturity was observed in YN, followed by JM, and finally, LX (Figure 2). Soil water consumption from the 100 to 120 cm soil layer in JM and LX did not differ with that of YN in 2017–2018 but were both lower than that of YN in 2018–2019. No significant differences were found in the soil water consumption of the 120–200 cm soil layer from anthesis to maturity among YN, JM, and LX in both years.

### Root Weight Density in the 0–100 cm Soil Layer

**FIGURE 3:**
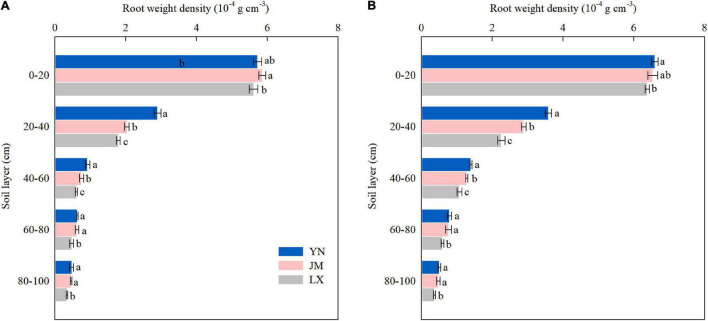
Root weight density in the 0–100 cm soil layer at anthesis of wheat varieties in the 2017–2018 **(A)** and 2018–2019 **(B)** growing seasons. YN, Yannong 1212; JM, Jimai 22; LX, Liangxing 99. The different letters in the figure indicate significant differences (Duncan’s test, *p* < 0.05). Vertical bars represent the SD of the means.

The RWD in the 0–40 cm soil layer accounted for 78.8–83.9% of the total RWD (i.e., in the entire 0–100 cm soil layer) in both years (Figure 3). In both years, the RWD of all three wheat varieties significantly decreased with an increase in soil depth at anthesis. The RWD from the 0 to 20 cm soil layer in YN did not differ with that of JM in both years but was higher than that of LX in 2018–2019. Compared to JM and LX, YN had greater RWD in the 20–60 cm soil layer in both years. The RWD in the 60–100 cm soil layer did not differ between YN and JM, which were both higher than that of LX.

### Root Morphology Characteristics

In the 0–20 cm soil layer, there were no significant differences in root length density and root diameter at 0 DAA among wheat varieties in both years (Table 2). YN and JM had greater root length density, root surface area density, and root diameter at 10 DAA than those of LX. Maximum values for root length density, root surface area density, and root diameter at 20 DAA were observed in YN, followed by JM with the lowest in LX.

**TABLE 2:** Root morphology characteristics of winter wheat in the 0–40 cm soil layer after anthesis of wheat varieties in the 2017–2018 and 2018–2019 growing seasons.

Year	Root traits	Treatments	0–20 cm soil layer			20–40 cm soil layer		
			0 DAA	10 DAA	20 DAA	0 DAA	10 DAA	20 DAA
2017–2018	Root length density (cm cm^–3^)	YN	1.39a	1.26a	1.09a	0.79a	0.70a	0.62a
		JM	1.40a	1.26a	1.03b	0.80a	0.68a	0.59b
		LX	1.39a	1.18b	0.88c	0.76b	0.62b	0.53c
	Root surface area density (mm^2^ cm^–3^)	YN	12.82ab	10.30a	8.21a	6.43a	5.37a	4.21a
		JM	12.99a	10.19a	7.24b	6.53a	5.04b	3.49b
		LX	12.72b	8.60b	5.52c	5.83b	4.21c	2.81c
	Root diameter (mm)	YN	0.29a	0.26a	0.24a	0.26a	0.25a	0.22a
		JM	0.30a	0.26a	0.22b	0.26a	0.24b	0.19b
		LX	0.29a	0.23b	0.20c	0.24b	0.22c	0.17c
2018–2019	Root length density (cm cm^–3^)	YN	1.47a	1.39a	1.17a	0.89a	0.81a	0.69a
		JM	1.47a	1.39a	1.08b	0.90a	0.79a	0.65b
		LX	1.46a	1.26b	0.94c	0.85b	0.70b	0.57c
	Root surface area density (mm^2^ cm^–3^)	YN	14.44a	12.52a	9.74a	7.71a	6.52a	5.13a
		JM	14.39a	12.47a	8.35b	7.81a	6.03b	4.52b
		LX	13.98a	10.33b	6.46c	6.69b	4.84c	3.63c
	Root diameter (mm)	YN	0.31a	0.29a	0.27a	0.28a	0.26a	0.24a
		JM	0.31a	0.29a	0.25b	0.28a	0.24b	0.22b
		LX	0.30a	0.26b	0.22c	0.25b	0.22c	0.20c

*YN, Yannong 1212; JM, Jimai 22; LX, Liangxing 99; DAA, days after anthesis. Values followed by a different letter are significantly different (Duncan’s test, p < 0.05) within the treatments in each year.*

In the 20–40 cm soil layer, YN and JM had higher root length density, root surface area density, and root diameter at 0 DAA than LX in both years (Table 2). The root surface area density at 10 DAA of YN showed 6.5% and 27.6%, and 8.1% and 34.7% higher than that of JM and LX, in 2017–2018 and 2018–2019, respectively. The root diameter at 10 DAA of YN was 4.2% and 8.3% greater than that of JM, and 13.6% and 18.2% higher than that of LX in 2017–2018 and 2018–2019, respectively. Root length density, root surface area density, and root diameter at 20 DAA were manifested in the following order: YN > JM > LX in both years.

### Root Senescence Characteristics

**FIGURE 4:**
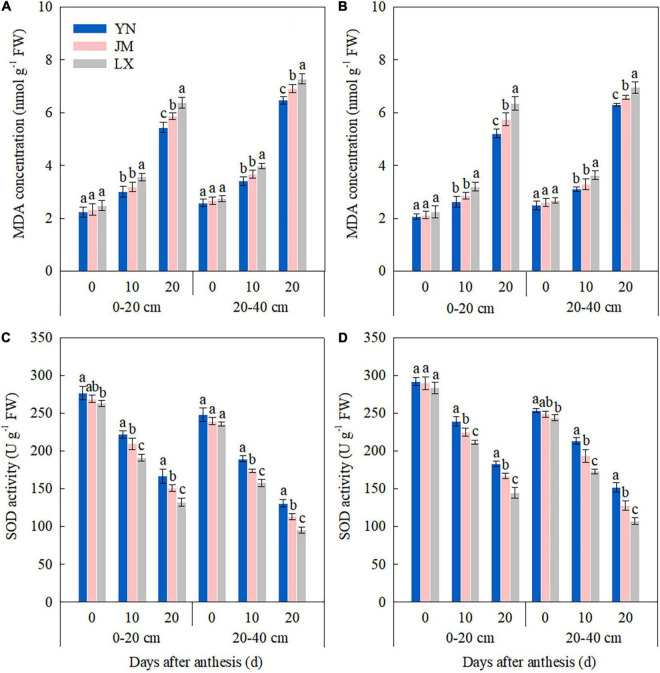
Malondialdehyde (MDA) concentration and superoxide dismutase (SOD) activity of root in 0–20 and 20–40 cm soil layers after anthesis in 2017–2018 **(A,C)** and 2018–2019 **(B,D)** growing seasons. YN, Yannong 1212; JM, Jimai 22; LX, Liangxing 99. The different letters in the figure indicate significant differences (Duncan’s test, *p* < 0.05). Vertical bars represent the SD of the means.

In the 0–20 cm soil layer, there was no significant difference in MDA concentration at 0 DAA among wheat varieties in both years (Figures 4A,B); however, compared with LX, YN, and JM had lower MDA concentration of root at 10 DAA. The MDA concentration of root at 20 DAA was manifested in the following order: LX > JM > YN. The MDA concentration trend in the 20–40 cm soil layer was similar to that at 0–20 cm. In the 0–20 cm soil layer, SOD activity at 0 DAA in YN did not differ to that of JM in both years but was higher than that of LX in 2017–2018 (Figures 4C,D). The SOD activity at 10 and 20 DAA were ranked in the order: YN > JM > LX. In the 20–40 cm soil layer, the differences of roots among wheat varieties were non-significant for SOD activity at 0 DAA in 2017–2018. The highest SOD activity of root at 10 and 20 DAA was obtained in YN, whereas the lowest was obtained in LX in both years.

### Root Triphenyl Tetrazolium Chloride Reduction Activity

In the 0–20 cm soil layer, root triphenyl tetrazolium chloride reduction activity (RTTC) at 0 DAA in YN did not differ with that of JM in both years but was higher than that of LX in 2018–2019 (Table 3). Compared with JM and LX, YN showed higher RTTC at 10 and 20 DAA. In the 20–40 cm soil layer, the differences of RTTC at 0 DAA among wheat varieties were non-significant in both years. Compared with JM and LX, YN had higher RTTC at 10 DAA by 14.7% and 34.9% in 2017–2018 and by 12.2% and 27.5% in 2018–2019, respectively. RTTC at 20 DAA was manifested in the following order: YN > JM > LX.

**TABLE 3:** Root triphenyl tetrazolium chloride (TTC) reduction activity of winter wheat in the 0–40 cm soil layer after anthesis of wheat varieties in the 2017–2018 and 2018–2019 growing seasons.

Year	Treatments	Root TTC reduction activity (μg g^–1^ h^–1^)					
		0–20 cm soil layer			20–40 cm soil layer		
		0 DAA	10 DAA	20 DAA	0 DAA	10 DAA	20 DAA
2017–2018	YN	73.7a	60.2a	45.2a	59.3a	52.2a	32.6a
	JM	72.4a	51.7b	37.2b	56.1a	45.5b	25.9b
	LX	70.8a	43.6c	32.0c	55.7a	38.7c	20.7c
2018–2019	YN	80.6a	61.7a	53.7a	61.7a	53.3a	39.1a
	JM	75.8ab	52.5b	48.0b	60.2a	47.5b	35.5b
	LX	71.4b	46.8c	42.2c	58.8a	41.8c	31.8c

*YN, Yannong 1212; JM, Jimai 22; LX, Liangxing 99; DAA, days after anthesis. Values followed by a different letter are significantly different (Duncan’s test, p < 0.05) within the treatments in each year.*

### Grain Yield and Water Use Efficiency

**FIGURE 5:**
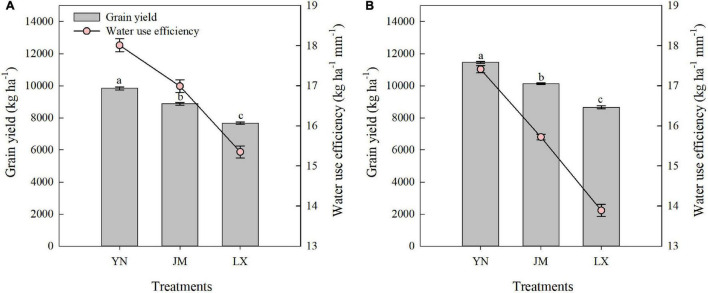
Grain yield and water use efficiency of wheat varieties in the 2017–2018 **(A)** and 2018–2019 **(B)** growing seasons. YN, Yannong 1212; JM, Jimai 22; LX, Liangxing 99. The different letters in the figure indicate significant differences (Duncan’s test, *p* < 0.05). Vertical bars represent the SD of the means.

Over the 2-year experimental period, the grain yields in YN were 10.8% and 13.1% higher than in JM, respectively, and 28.3% and 32.3% higher than in LX, respectively (Figure 5). In both years, the WUE was manifested in the following order: YN > JM > LX.

## Discussion

The wheat crop is more sensitive to a water deficit during the reproductive stage than the vegetative growth stage (Ma et al., 2019). Harmsen et al. (2009) indicated that crop evapotranspiration was significantly correlated with crop production. Water consumption in winter wheat reaches peaks during the heading and filling stages to maintain normal growth and grain production (Jin et al., 2017). In this study, on average, ET accounted for 26.9–37.0%, 26.9–27.0%, and 36.1–46.2% of ET_C_ from planting to jointing, jointing to anthesis, and anthesis to maturity stages, respectively (Table 1). Compared to YN, JM, and LX with lower yields both had higher CP from planting to jointing and from jointing to anthesis but a lower CP from anthesis to maturity stages (Figure 5 and Table 1). The significant increases in CP from anthesis to maturity stages of JM and LX in the following year may be due to the monthly precipitation fluctuations (Supplementary Figure 1) because the water requirement of winter wheat can be influenced by interannual precipitation variability (Zhao et al., 2020). A previous study showed that wheat yield and WUE in the water shortage area could increase by increasing post-anthesis water use amount and ratio via a reasonable irrigation strategy (Xu et al., 2018). In this study, compared with JM and LX, YN showed lower CP from planting to anthesis but had higher ET and CP from anthesis to maturity under the same irrigation measures (Table 1), indicating that the post-anthesis ET and CP should be considered when selecting varieties for cultivation in the 3HP. Further, high-yielding YN can coordinate pre- and post-anthesis water use and improve the water consumption ratio after anthesis, which is beneficial to the formation of grain yield.

As precipitation cannot meet wheat water demand in 3HP, available soil water is required as an additional sources of water supply (Yang M. D. et al., 2020; Shirazi et al., 2022). The differences in the root system of wheat influenced the soil water extraction during the whole growth season of wheat (Středa et al., 2012). Over 85% consumption of the available soil water stored in the 0–50 cm layer at planting occurs due to high root density and evaporation (Shunqing et al., 2003). Wheat varieties with a higher probability of extracting water from deeper soil profiles at the vegetative phase could provide an early indication of plant productivity and lead to higher yield (Corneo et al., 2018). Thapa et al. (2020) showed that under limited irrigation conditions, water used from deeper soil layers significantly contributed to wheat yield compared to water only drawn from the shallower profile, especially in the late growing season. In this study, the differences of soil water extraction from anthesis to maturity among wheat varieties were focused in the upper 100 cm soil profile in both seasons (Figure 2). The higher ET and CP during anthesis to maturity shown by YN was attributed to the additional soil water consumption in the 0–100 cm soil layer (Table 1 and Figure 2). This could be partly credited to the greater RWD of YN in the 20–60 cm soil layer at anthesis (Figure 3), because root distribution can greatly affect water extraction by crops, and root dry weight in deep soil layer had positive relationships with grain yield and WUE (Kang et al., 2014; Fang et al., 2017). In this study the vertical distribution of RWD in YN was not only conducive to the use of water in the 20–60 cm soil layer but was also beneficial to promoting the use of water from the deeper soil layers (60–100 cm), contributing to the improved water use pattern of YN. Measurements for the 0–20 cm soil layer have not been included in this relation in this study, which, due to the extractable water, may include the evaporative loss from the soil surface.

Most of the root distribution was in the top 0–40 cm layer of the soil profile (Fang et al., 2021). Our results supported the result that 78.8–83.9% of the total RWD (i.e., in the entire 0–100 cm soil layer) was distributed in the 0–40 cm soil layer (Figure 3). The enhancement of shallow root growth for high-yielding wheat production has been proposed for its role in the absorption of soil nutrients concentrated in the upper layers and the capture of precipitation (Hermanska et al., 2015; Becker et al., 2016). Fang et al. (2021) reported that wheat varieties with large root biomass and root length density in the 0–40 cm soil layer had negative effects on post-anthesis soil water use under rainfed conditions in the semi-arid area on the Loess Plateau. However, Qin et al. (2019) indicated that modern wheat varieties are better adapted on irrigated land than older varieties, due to the increased root biomass at shallow depth assisting water uptake to support greater shoot biomass and grain yield. While root biomass is not directly equivalent to root surface area, it could be assumed that a more extensive root systems could have greater biomass as well as an increased surface area (McGrail and McNear, 2021). In this study compared to JM and LX, high-yielding YN had more water consumption after anthesis and showed better root morphology characteristics in the topsoil (0–40 cm) during mid grain filling stage (root length density, root surface area density, and root diameter at 20 DAA) (Table 2), indicating that wheat varieties with better root morphology characteristics during mid grain filling stage could contribute to the increases in soil water absorption and wheat yield. This result is consistent with the findings of Feng et al. (2017), who showed that better root morphology characteristics were beneficial for high yield.

Hu et al. (2018) reported that under water stress conditions, the drought-tolerant wheat genotype, JM-262, showed higher antioxidase activity and a greater root system to uptake more water at the seedling stage. In sunflower (*Helianthus annuus* L.), root senescence during the grain filling stage precedes the canopy senescence that is closely links with yield formation (Lisanti et al., 2013). Our results showed that compared with JM and LX, YN had higher SOD and RTTC at 10 and 20 DAA both in 0–20 and 20–40 cm soil layers (Figure 4 and Table 3), indicating that selecting high-yielding YN with alleviated root senescence in the upper soil layers during mid grain filling period could also contribute to the increases in soil water absorption post-anthesis and wheat yield. Because Man et al. (2016) reported that root TTC reduction activity and root SOD activity post-anthesis were strongly positively correlated with soil water consumption after anthesis, grain yield and WUE. And studies performed in rice (Liu et al., 2021) and maize (Wang et al., 2019) have been clearly demonstrated that delayed root senescence can contribute to the yield enhancement by optimizing resource acquisition from the soil.

Ensuring water use after anthesis largely improved dry matter production, which accelerated grain formation, and hence, grain yield (Xu et al., 2018). Thapa et al. (2020) showed that wheat with a grain yield of 4,807 kg ha^–1^ extracted 165 mm of stored soil water, while only 70 mm of stored soil water was extracted in wheat with a grain yield of 2,933 kg ha^–1^. Additional consumption of 10.5 mm soil water after anthesis has been shown to increase grain yield by 620 kg ha^–1^ under moderate post-anthesis stress (Kirkegaard et al., 2007). Our study showed that compared to JM and LX, additional consumption of 22.7–44.6 mm soil water after anthesis in YN increased grain yield by 960–2797 kg ha^–1^ (Figures 1, 5). The WUE in YN were 6.0% and 10.8% higher than in JM, respectively, and 17.3% and 25.3% higher than in LX, respectively, in 2017–2018 and 2018–2019. Besides producing the same grain yield from less water resources, an increased WUE in crop production can also be achieved through increased grain yield by cultivar replacement (Zhang et al., 2017; Yan et al., 2021). In this study, the increase in WUE of YN was attributed to the increased grain yield, because YN increased SWC_AM_ and obtained the highest ET_C_ (Figure 1 and Table 1).

## Conclusion

The high-yielding wheat variety YN with high WUE showed higher the ET and CP after anthesis and extracted more soil water in the 0–100 cm soil layer post-anthesis. Moreover, compared with JM and LX, YN obtained larger RWD in the 20–60 cm soil layer at anthesis, better root morphology characteristics, greater root antioxidant enzyme activity and higher RTTC in the 0–40 cm soil layer during mid grain filling stage. Thus, improving root development [shown as larger root distribution in 20–60 cm soil profile, improved root morphology traits and alleviated root senescence in the topsoil (0–40 cm) during the mid grain filling phase] helps to increase soil water extraction post-anthesis and should be considered as a critical strategy for breeding wheat varieties with high yield and high WUE.

## Data Availability Statement

The raw data supporting the conclusions of this article will be made available by the authors, without undue reservation.

## Author Contributions

YS and ZY conceived and design the study and revised the manuscript. XZ and PL performed the experiments. XZ analyzed the data and wrote the manuscript. All authors have made good contributions to this work.

## Conflict of Interest

The authors declare that the research was conducted in the absence of any commercial or financial relationships that could be construed as a potential conflict of interest.

## Publisher’s Note

All claims expressed in this article are solely those of the authors and do not necessarily represent those of their affiliated organizations, or those of the publisher, the editors and the reviewers. Any product that may be evaluated in this article, or claim that may be made by its manufacturer, is not guaranteed or endorsed by the publisher.
