# Supplementary material for: Differences in Water Consumption of Wheat Varieties Are Affected by Root Morphology Characteristics and Post-anthesis Root Senescence
Source: Front Plant Sci. 2022 Jan 31;12:814658. doi: 10.3389/fpls.2021.814658 (PMC8841790; doi:10.3389/fpls.2021.814658)
Supplement: Supplementary file 1 [file Data_Sheet_1.docx]

**Supplementary Material for**

**Differences in water consumption of wheat varieties are affected by root morphology characteristics and post-anthesis root senescence**

Xuejiao Zheng, Zhenwen Yu, Yu Shi*, Peng Liang

**This file includes:**

Figure S1 and Table S1.


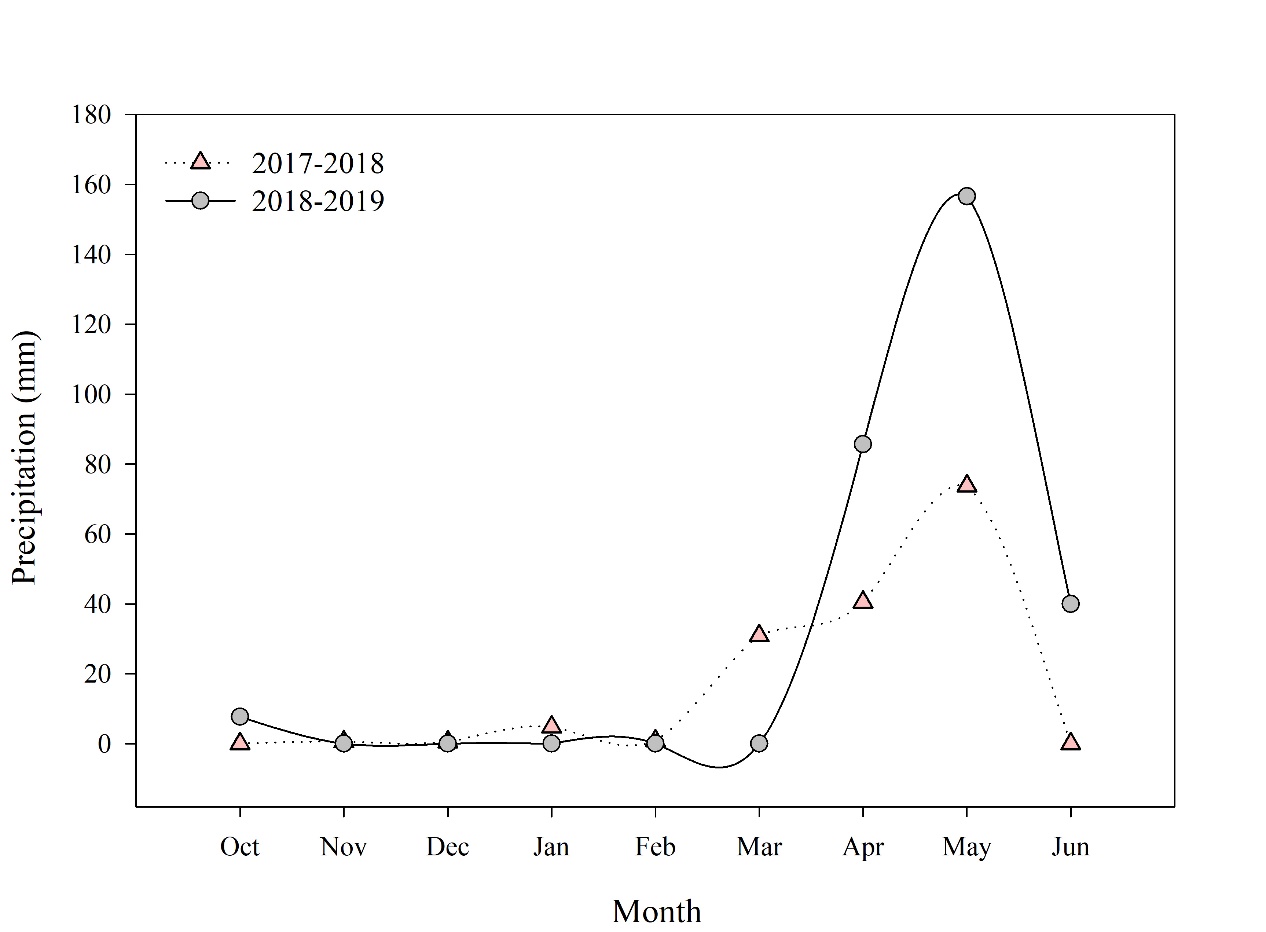


**Fig. S1** Precipitation during the wheat growing seasons in 2017­–2018 and 2018­–2019.

**Table S1** Soil bulk density and field capacity in the 0–200 cm soil layer before sowing in the experimental plots

| Soil layer | 2017­–2018 growing season | |  | 2018­–2019 growing season | |
| --- | --- | --- | --- | --- | --- |
|  | Soil bulk density | Field capacity |  | Soil bulk density | Field capacity |
| cm | (g cm^-3^) | (%) |  | (g cm^-3^) | (%) |
| 0–20 | 1.42 | 27.25 |  | 1.41 | 29.54 |
| 20–40 | 1.58 | 23.31 |  | 1.56 | 25.44 |
| 40–60 | 1.56 | 26.09 |  | 1.54 | 27.69 |
| 60–80 | 1.59 | 24.75 |  | 1.57 | 25.35 |
| 80–100 | 1.62 | 23.77 |  | 1.63 | 24.58 |
| 100–120 | 1.63 | 23.40 |  | 1.64 | 24.22 |
| 120–140 | 1.64 | 23.80 |  | 1.64 | 23.54 |
| 140–160 | 1.63 | 23.74 |  | 1.65 | 23.64 |
| 160–180 | 1.64 | 23.66 |  | 1.64 | 23.71 |
| 180–200 | 1.64 | 23.61 |  | 1.65 | 23.08 |
